# Discovery of human-like L-asparaginases with potential clinical use by directed evolution

**DOI:** 10.1038/s41598-017-10758-4

**Published:** 2017-08-31

**Authors:** Coraline Rigouin, Hien Anh Nguyen, Amanda M. Schalk, Arnon Lavie

**Affiliations:** 1 Department of Biochemistry and Molecular Genetics, University of Illinois at Chicago, Chicago, Illinois United States of America; 2 The Jesse Brown VA Medical Center, Chicago, Illinois United States of America

## Abstract

L-asparaginase is a chemotherapy drug used to treat acute lymphoblastic leukemia (ALL). The main prerequisite for clinical efficacy of L-asparaginases is micromolar K_M_ for asparagine to allow for complete depletion of this amino acid in the blood. Since currently approved L-asparaginases are of bacterial origin, immunogenicity is a challenge, which would be mitigated by a human enzyme. However, all human L-asparaginases have millimolar K_M_ for asparagine. We recently identified the low K_M_ guinea pig L-asparaginase (gpASNase1). Because gpASNase1 and human L-asparaginase 1 (hASNase1) share ~70% amino-acid identity, we decided to humanize gpASNase1 by generating chimeras with hASNase1 through DNA shuffling. To identify low K_M_ chimeras we developed a suitable bacterial selection system (*E. coli* strain BW5Δ). Transforming BW5Δ with the shuffling libraries allowed for the identification of several low K_M_ clones. To further humanize these clones, the C-terminal domain of gpASNase1 was replaced with that of hASNase1. Two of the identified clones, 63_N_-h_C_ and 65_N_-h_C_, share respectively 85.7% and 87.1% identity with the hASNase1 but have a K_M_ similar to gpASNase1. These clones possess 100–140 fold enhanced catalytic efficiency compared to hASNase1. Notably, we also show that these highly human-like L-asparaginases maintain their *in vitro* ALL killing potential.

## Introduction

L-asparaginases, enzymes that catalyze the hydrolysis of L-asparagine (Asn) to L-aspartic acid (Asp) and ammonia, are currently a mainstay treatment for several hematological malignancies. The discovery of this therapeutic modality was made serendipitously in 1953 by Kidd who reported that the serum from guinea pigs could kill transplanted lymphomas in mice^1^. The factor responsible for the cell killing ability of the guinea pig serum was identified by Broome in 1961 as an enzyme with L-asparaginase activity^2^.

As the identification of the guinea pig enzyme behind the L-asparaginase activity proved difficult, enzymes from other source were sought. *E. coli* contains three L-asparaginases, referred to as type I, II, and III. It is only the *E. coli* type II enzyme that possesses the required property for clinical use (brand name Elspar)^3^ – namely a K_M_ for Asn in the low micromolar range that is able to deplete the Asn in the blood^4^. Since becoming FDA-approved in 1978, L-asparaginase therapy has become key for inducing remission for acute lymphoblastic leukemia (ALL).

A major disadvantage to the use of bacterial enzymes as therapeutics is their immunogenicity, which can pose a direct threat to the patient due to hypersensitivity reactions, up to anaphylactic shock. Moreover, generated antibodies can inactivate and clear the enzyme drug, thus reducing or even eliminating its effectiveness^5^. Methods have been developed to reduce these severe side effects, such as conjugation of the *E. coli* enzyme with polyethylene glycol^6^ (brand name Oncaspar, FDA-approved in 1994, first-line treatment in 2006) or the identification of enzymes from different sources, specifically from the bacterium *Erwinia chrysanthemi* (brand name Erwinaze, FDA-approved in 2011). However, these alternative L-asparaginase preparations still suffer from being immunogenic^7, 8^.

We propose that replacing the bacterial L-asparaginases with human-like enzymes would reduce the immunological problems associated with current L-asparaginase therapy. This concept is supported by the evidence from recombinant human enzymes being used extensively as replacement therapy for lysosomal storage disorders such as Gaucher, Fabry, and Pompe diseases^9^. Unfortunately, the wild-type versions of known human L-asparaginases are not suitable replacements for the clinically used bacterial enzymes since they possess a very high K_M_ value for Asn^10–12^. Given the physiological concentration of Asn in blood (~50 μM)^4^, the enzyme must have an Asn K_M_ in the low micromolar range to be clinically relevant. Therefore, we initiated directed evolution strategies geared at engineering a human L-asparaginase to acquire a low Asn K_M_ value.

Directed evolution, or the process to mimic natural evolutionary processes in the laboratory, is widely used to improve enzyme properties. However, modifying a given candidate to attain a desired property is difficult given the large protein-sequence space and the operational limit of any experimental screen and selection methods. A number of reviews have discussed directed evolution methodologies^13–16^. Thanks to advances in library generation, screening techniques and foremost a better understanding of mechanisms of natural protein evolution, researchers have been able to obtain large improvements in the evolved catalytic activity (relative to the starting point, and in absolute k_cat_/K_M_ values)^17, 18^. Bar-Even *et al*.^19^ showed that both evolutionary selection pressures and physicochemical constraints could shape the kinetic parameters of enzymes and that the catalytic efficiency of some enzymes toward their natural substrates could be increased in many cases by natural or laboratory evolution.

The first step of the process towards a human-like L-asparaginase with micromolar K_M_ for the substrate Asn is to choose the L-asparaginase to be engineered. We selected the human enzyme annotated as 60 kDa lysophospholipase (UniProt entry Q86U10) since it is homologous to the *E. coli* type I & II enzymes (type I is cytoplasmic and has a high K_M_ for asparagine, type II is periplasmic and has a low K_M_). Since the K_M_ for Asn of this human L-asparaginase is in the millimolar range^10, 11^, similar to that of the *E. coli* type I, we refer to this enzyme as human L-asparaginase 1 (hASNase1).

Generating genetic diversity (i.e. a large library of gene variants) can be accomplished by random or saturation mutagenesis protocols or alternatively by DNA family shuffling protocols. For the DNA family shuffling approach^20^, one would recombine gene fragments of hASNase1 with those from homologous low K_M_ enzymes to make chimeras. For this approach to work, the homologous genes must be relatively similar on the DNA level to allow for recombination. As noted previously, the clinically used *E. coli* type II enzyme has low homology to hASNase1 (26% amino acid sequence identity), limiting its use as a fragment source for the DNA shuffling approach. However, we recently identified a guinea pig L-asparaginase that has a micromolar K_M_ for asparagine and that enzyme is ~70% identical to hASNase1^11^. Therefore, we used the gene of this guinea pig enzyme to prepare the libraries for the DNA family shuffling protocol.

The second step of the process was to develop a directed evolution selection system. Since the L-asparaginase reaction yields two products, aspartate and ammonia, conceptually one can develop a selection strategy that requires the presence of either of these molecules. In this work, we describe selection systems that exploit either of these L-asparaginase reaction products.

The third step of the directed evolution process is to amplify and analyze the variants selected to display the desired property. Here we describe how by following these steps we discovered variants of hASNase1 that are over 90% identical in amino acid sequence to the wild-type enzyme, but have as much as 100-fold reduced K_M_ value for Asn. Most importantly, these variants have comparable kinetic properties to the clinically used L-asparaginases and equivalent *in vitro* cell killing potency to the gpASNase1, making them good candidates for the development of potentially less immunogenic replacements of the FDA-approved bacterial L-asparaginases.

## Results

### Development of the selection systems

To create a selection system for L-asparaginase activity, a bacterial strain whose growth is dependent on any product of the enzymatic reaction is required. Since L-asparaginase catalyzes the hydrolysis of Asn into Asp and ammonia, two selections systems were developed and tested: one employing a bacterial strain dependent on the reaction product ammonium as the sole nitrogen source and the other uses a strain auxotroph for Asp.

#### Strain BW2Δ: dependence on L-asparaginase reaction for nitrogen source

**Figure 1:**
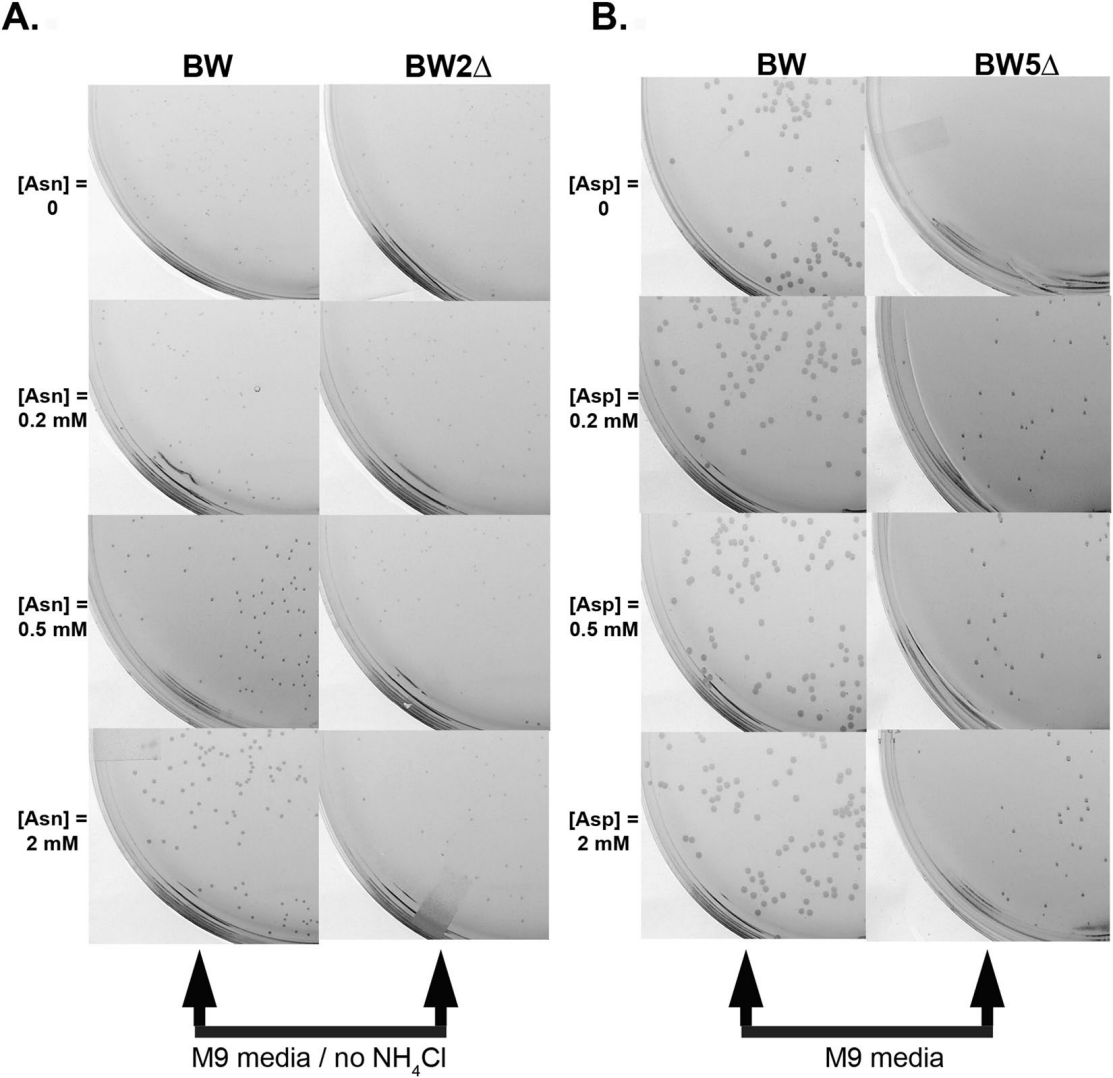
Development of an *E. coli* strain dependent on the L-asparaginase reaction. (**A**) Dependence on the ammonium produced by the L-asparaginase reaction: shown are colony formation of the strains BW after 48 h and BW2Δ after 72 h in complete minimal medium M9 agar that lacks ammonium chloride and was supplemented with increasing concentrations of Asn (0, 0.2, 0.5 and 2 mM) as the sole source of nitrogen. Whereas the BW strain can obtain the required nitrogen from hydrolyzing Asn, forming many colonies after only 48 h, the BW2Δ strain forms fewer and smaller colonies even after 72 h. (**B**) Dependence on the Asp produced by the L-asparaginase reaction: shown are colony formation of the strains BW after 48 h and BW5Δ after 72 h in complete minimal medium M9 agar supplemented with increasing concentrations of Asp (0, 0.2, 0.5 and 2 mM). Whereas growth in agar of the BW strain is not dependent on Asp, growth of the BW5Δ exhibits a dose response to this amino acid.

All life forms require a nitrogen source. For *E. coli* grown in a minimal media such as M9, the nitrogen source is usually obtained in the form of NH_4_Cl salt. However, in the absence of NH_4_Cl, we verified that the *E. coli* BW strain used in this study could grow using 2 mM Asn in the media (Fig. 1A, bottom left panel). This demonstrates that *E. coli* can use Asn as a nitrogen source through the activity of its endogenous L-asparaginases. To make the BW *E. coli* strain dependent on the NH_4_Cl produced by exogenous L-asparaginase activity, two endogenous L-asparaginase genes (ansA and ansB) were deleted from the *E. coli* BW parental strain, which we then referred to as BW2Δ (we omitted deleting the third endogenous L-asparaginase (gene name iiiA), since it has a very high K_M_ value for Asn). Indeed, under the experimental conditions, even with 2 mM Asn in the media, growth of the BW2Δ strain was greatly impaired (Fig. 1A, bottom right panel).

#### Strain BW5Δ: dependence on L-asparaginase reaction for aspartate

*E. coli* can generate Asp by hydrolyzing Asn (the L-asparaginase reaction) or by an aminotransferase reaction. Indeed, the parental BW strain grows well in M9 media independently and with no effect by Asp supplementation (Fig. 1B, BW column). To create an *E. coli* strain auxotrophic for Asp, we knocked out all three endogenous L-asparaginase genes (ansA, ansB, and iiiA) in addition to the two relevant aminotransferase genes (aspC and tyrB) – see Methods. The strain with these five genes deleted is referred to as strain BW5Δ. To ensure that the strain BW5Δ acquired Asp auxotrophy, we tested growth in minimal media M9 with and without Asp supplementation. We found that without supplementing the media with Asp, BW5Δ could not grow (Fig. 1B, BW5Δ top line panel). However, growth was observed in the Asp supplemented conditions.

#### Use of the BW2Δ and BW5Δ strains to select clones expressing L-asparaginase

To investigate whether these bacterial strains can be used as selection systems for L-asparaginase activities and foremost whether they would allow the differentiation between L-asparaginases based on their K_M_ property, we followed the growth of the BW2Δ and BW5Δ strains expressing either an L-asparaginase with a low K_M_ or an L-asparaginase with a high K_M_. Human L-asparaginase type I (hASNase1), the protein target to be evolved, is characterized by a K_M_ for Asn in the millimolar range^10, 11^ and was thus used as the high K_M_ L-asparaginase. The guinea pig L-asparaginase I (gpASNase1) was characterized in our lab having a K_M_ for Asn in the micromolar range^11^ and was thus used as the low Km L-asparaginase. Both genes encoding the respective L-asparaginase were cloned into the pBAD vector in order to have well-controlled protein expression.

**Figure 2:**
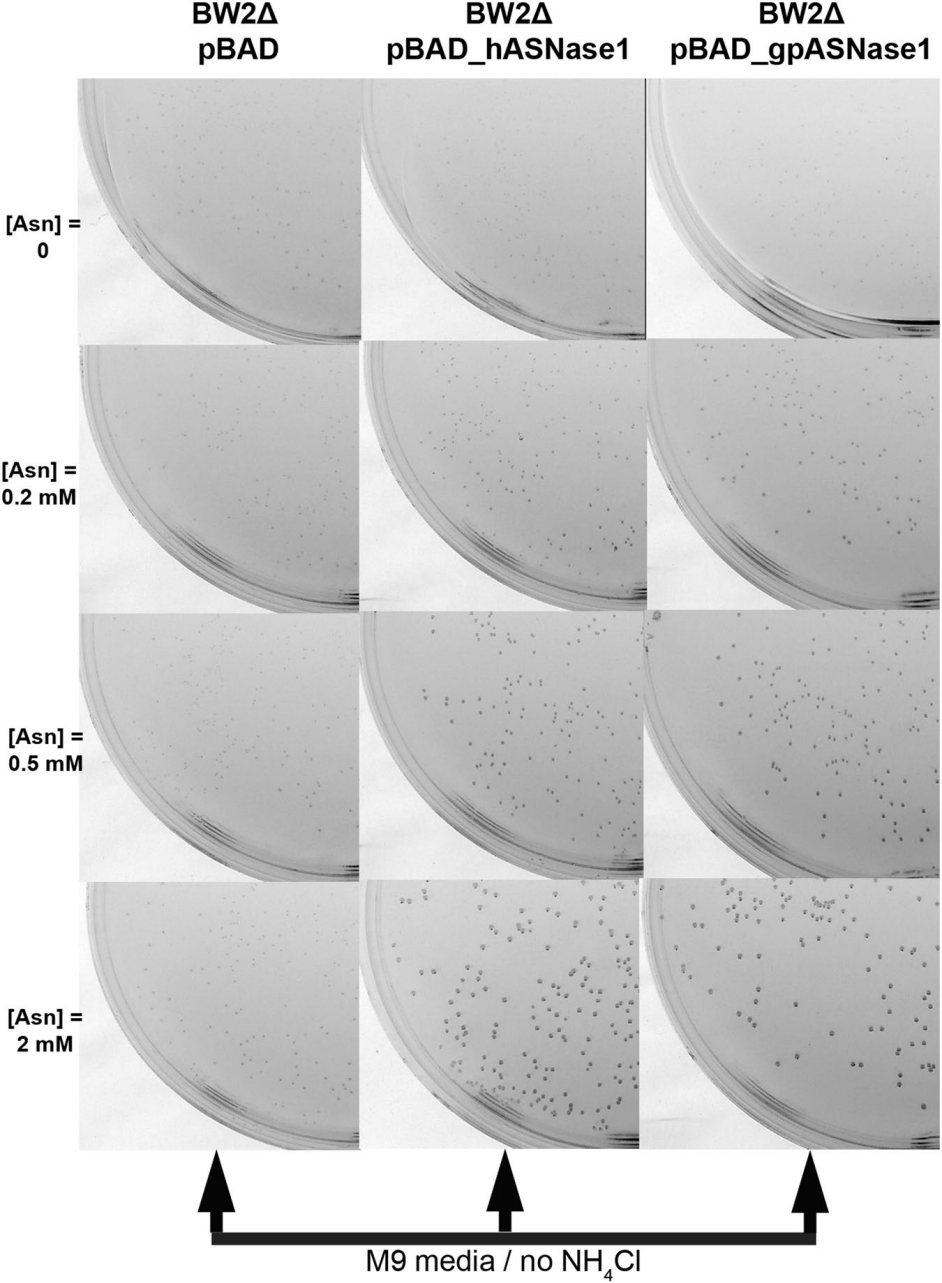
Colony formation of the BW2Δ strain is dependent on the ammonium product of the L-asparaginase reaction but not on the Asn K_M_ of the enzyme. Colony formation at 37 °C after 72 h of the control strain BW2Δ pBAD and the L-asparaginase expressing strains: BW2Δ gpASNase1 (low Km enzyme) and BW2Δ pBAD_hASNase1 (high Km enzyme) on minimal medium M9 agar supplemented with increasing concentration of Asn (0, 0.2, 0.5 and 2 mM) and 0.02% of arabinose. Whereas expression of both L-asparaginases promotes colony formation, both the low and high Km enzymes do this with similar efficiency.

In the first screening system, which is based on the L-asparaginase reaction supplying the sole source of nitrogen, BW2Δ was transformed with pBAD (control vector), pBAD_hASNase1 (high K_M_ enzyme) or pBAD_gpASNase1 (low K_M_ enzyme). The transformed cells were grown in M9 medium lacking NH_4_Cl but supplemented with an increasing concentration of Asn. The expectation was that a low Asn concentration would preferentially promote the growth of the bacteria carrying the pBAD_gpASNase1 plasmid, coding for the low K_M_ enzyme. Results show that growth of both BW2Δ pBAD_hASNase1 and pBAD_gpASNase1 did in fact depend on the concentration of Asn (i.e. enhanced concentration of Asn leading to better growth) - Fig. 2. However, per Asn concentration, there was not a significant difference of growth between BW2Δ pBAD_hASNase1 and pBAD_gpASNase1. (e.g. at 0.2 mM Asn, BW2Δ bacteria that express the low Km gpASNase1 enzyme did not form significantly more or bigger colonies than bacteria that express the high Km hASNase1 enzyme). In sum, this screening system was found to be not suitable for discriminating between L-asparaginases that differ in their Asn K_M_ value.

**Figure 3:**
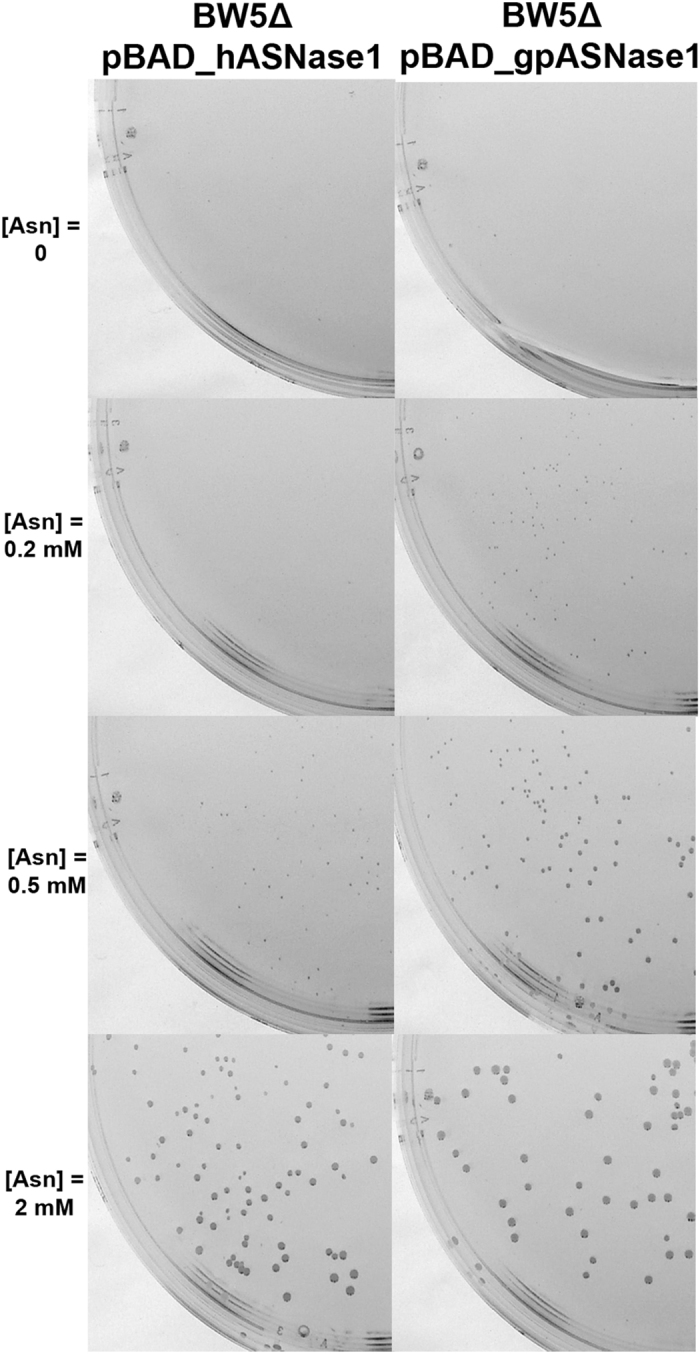
Colony formation of the BW5Δ strain is dependent on the Asp product of the L-asparaginase reaction and on the Asn Km of the enzyme. Colony formation at 37 °C after 72 h of the L-asparaginase expressing strains BW5Δ pBAD_gpASNase1 and BW5Δ pBAD_hASNase1 on minimal medium M9 supplemented with increasing concentrations of Asn (0, 0.2, 0.5 and 2 mM) and 0.02% arabinose. More and larger colonies are produced in the bacteria expressing the low K_M_ gpASNases1 enzyme in an Asn concentration dependent manner.

In the second screening system, which is based on Asp auxotropy, BW5Δ was similarly transformed with pBAD, pBAD_hASNase1 or pBAD_gpASNase1. The transformed cells were grown in complete M9 media supplemented with increasing concentration of Asn. Growth of BW5Δ pBAD_hASNase1 and pBAD_gpASNase1 were found to be dependent on the concentration of Asn (Fig. 3). Interestingly, at 2 mM Asn, which represents a concentration at which the guinea pig enzyme is saturated with substrate (K_M_ = 50 µM) and the human enzyme is only partially saturated (K_M_ = 3,500 µM), hASNase1 and gpASNase1 transformed BW5Δ strains show similar growth. In other words, at this relatively high Asn concentration, the BW5Δ strain cannot be used to distinguish between those bacteria that express a low Km L-asparaginase to those that express a high K_M_ enzyme. In contrast, at lower concentrations of the substrate Asn, only BW5Δ pBAD_gpASNase1 is capable of growing. Indeed, at 0.2 mM Asn, a concentration of Asn well below the K_M_ of hASNase1 but above the K_M_ of gpASNase1, colonies of BW5Δ pBAD_gpASNase1 developed whereas BW5Δ pBAD_hASNase1 could not grow (Fig. 3). Noteworthy, we found this growth difference to be independent of enzyme expression level since no change in growth was noticed when arabinose (the inducer for protein expression) was used in the range 0.0002–0.2% (data not shown). Taken together, the results suggest that the difference in growth seen between BW5Δ expressing hASNase1 and BW5Δ expressing gpASNase1 on minimal media plates directly reflects the K_M_ of the respectively expressed L-asparaginase.

The conclusion from this initial set of experiments is that the screen based on the L-asparaginase reaction supplying the nitrogen (using the BW2Δ strain) does not differentiate well enough between high and low K_M_ enzymes, whereas the screen based on the L-asparaginase reaction supplying the amino acid Asp (using the BW5Δ strain) does indeed discriminate between enzymes with differing affinities to Asn - at low Asn concentration, only the bacteria expressing the low K_M_ L-asparaginase forms colonies. Hence, all further selection work aimed at discovering a human L-asparaginase variant that has acquired a low K_M_ was performed with the BW5Δ *E. coli* strain.

### Generation of genetic diversity

#### Random and Combinatorial Active-site Saturation Test (CAST) mutagenesis

Our initial strategy for generating genetic diversity in hASNase1 was to employ random mutagenesis (on the full-length sequence and C-truncated construct) by error prone PCR^21^. This method allows the introduction of mutations randomly along the gene; the protocol used (see Methods) allowed a mutation rate of 0.2%. It has been largely used in directed evolution and has proven its efficiency in improving enzyme properties, particularly when the relation between the structure and the function of the enzyme is not known. CAST mutagenesis^22^ on the other hand makes use of available structural information to select specific residues in the active site that are to be mutated by site-directed mutagenesis. Both of these methods were performed on the hASNase1 gene. The CAST method was based on a homology model of hASNase1 that we generated from the crystal structure of gpASNase1^11^. Eight libraries of simultaneous randomization at two amino or three acid positions were generated (Figure S1). Libraries of hASNase1 mutants created by these two methods were used to transform the BW5Δ strain and plated on minimal media supplemented with a low Asn concentration (0.2 mM) to select improved variants. Disappointedly, no active variants were selected using these methods.

#### DNA family shuffling

**Figure 4:**
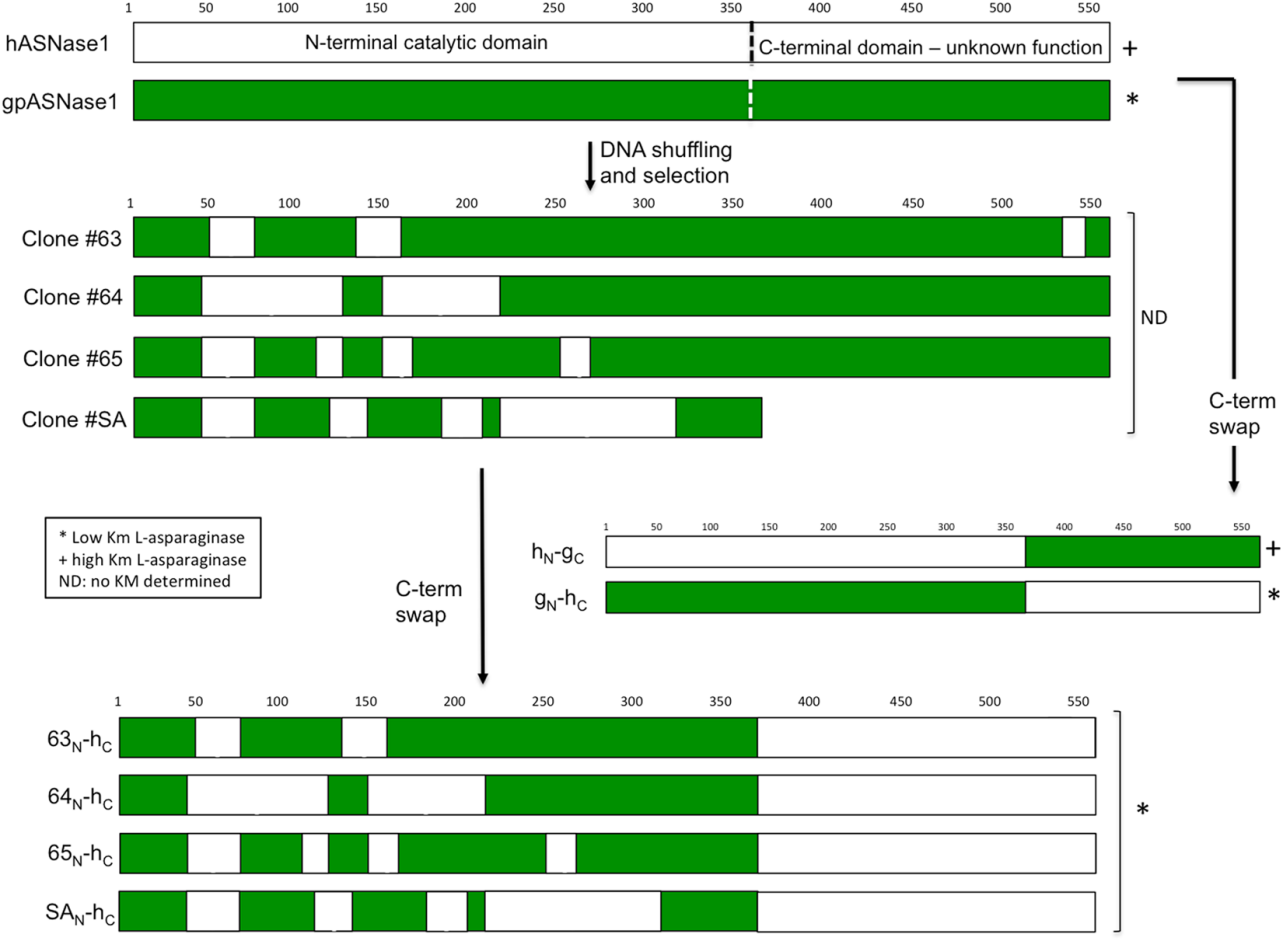
Schematic representation of the clones obtained from the DNA shuffling process and C-terminal domain swapping. hASNase1 sequences are depicted in white, gpASNase1 in green. Clones isolated from selection (#63, 64, 65 and SA) or generated by C-terminal swapping (63-hC, 64-hC, 65-hC and SA-hC) are a shuffle between hASNase1 (seen in white) and gpASNase1 (seen in green) sequences.

DNA family shuffling is an alternative method for generating genetic diversity. The protein sequences of hASNase1 (Figure S2, SEQ ID No1) and gpASNase1 (Figure S3, SEQ ID No2) contain 573 and 565 amino acids, respectively and are 69.8% identical on the amino acid level (differing by 170 amino acids). In our laboratory, we have worked with the synthetic codon-optimized version of both genes (Figure S4, SEQ ID No3 and Figure S5, SEQ ID No4). The two genes show 75% identity on the DNA level. As mentioned above, hASNase1 has a K_M_ of 3.5 mM for Asn, whereas the K_M_ of the guinea pig enzyme was determined to be 50 µM (Table 1). The DNA shuffling method was used to recombine the two L-asparaginases in order to obtain a chimera that displays the low K_M_ of the guinea pig but with as high as possible amino acid homology with the human enzyme. The shuffled fragments were then cloned into the pBAD vector. The resulting chimeric library was used to transform the BW5Δ strain. The presence of mutants possessing a low K_M_ was discovered using the selection protocol as described in the Methods section. Four clones (#63, #64, #65 and #SA) were isolated from M9 plates at an Asn concentration of 0.2 mM. Sequence analyses of these clones revealed a shuffling pattern with recombination events occurring predominantly in the N-terminal (i.e. catalytic) domain; one of the selected clones (#SA) carried a mutation that introduced a premature stop codon (STOP) that was beyond the catalytic domain (Fig. 4).

**Table 1:** Kinetic properties of the purified L-asparaginases used in this study.

Enzyme name	# of residues	% identity to hASNase1	% similarity to hASNase1	k_cat_ (sec^−1^)	K_M_ (µM)	k_cat_/K_M_ (sec^−1^µM^−1^)	k_obs_ (sec^−1^) @50 µM
hASNase1*	573	100	100	17 ± 0.8	3,500 ± 300	0.005	ND
ansB	326	26.9	58.5	48 ± 1	11 ± 1	4.4	41 ± 0.3
gpASNase1	565	69.8	88.6	41 ± 2	50 ± 7	0.8	20 ± 1
g_N_-h_C_	571	83.4	94.9	24 ± 1	35 ± 4	0.7	14 ± 1
h_N_-g_C_	567	86.5	93.7	14 ± 0.5	3,800 ± 120	0.004	ND
63_N_-h_C_	571	85.7	95.6	32 ± 0.6	47 ± 3	0.7	17 ± 0.5
64_N_ -h_C_	571	91.1	96.7	60 ± 2	202 ± 17	0.3	10 ± 1
65_N_ -h_C_	571	87.1	95.8	40 ± 1	74 ± 5	0.5	17 ± 1
SA_N_ -h_C_	571	91.6	97.4	32 ± 2	165 ± 19	0.2	6 ± 1

*Kinetic parameters using the Hill-equation (n = 2.1).

ND: not determined.

#### Swapping of the C-terminal domain

**Figure 5:**
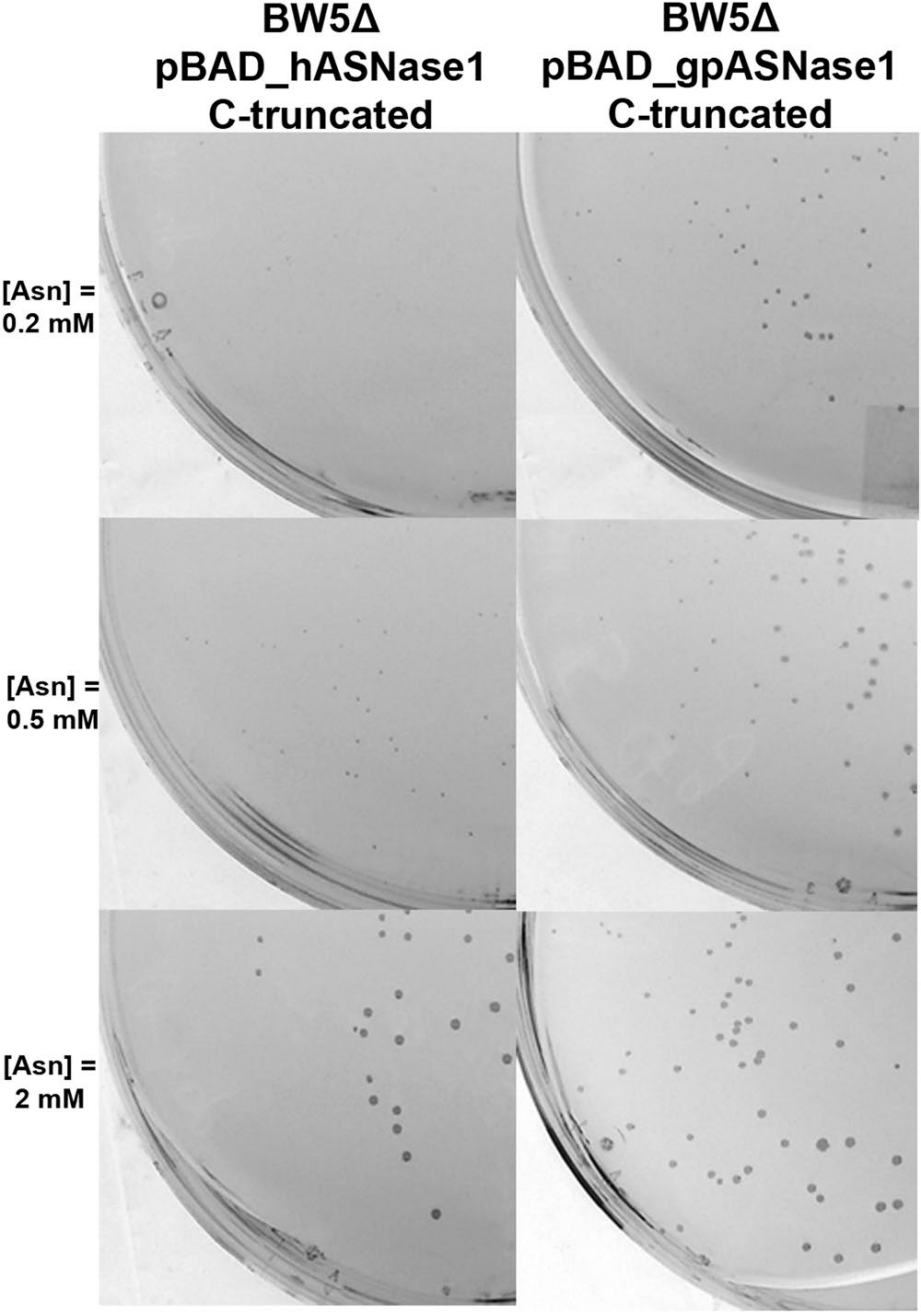
The C-terminal domain of the mammalian L-asparaginases has minimal influence on the N-terminal catalytic domain. Colony formation at 37 °C after 72 h of C-truncated L-asparaginase expressing strains BW5Δ pBAD_C-truncated_gpASNase1 and BW5Δ pBAD_C-truncated_hASNase1 on minimal medium M9 supplemented with increasing concentrations of Asn (0, 0.2, 0.5 and 2 mM) and 0.02% arabinose. The growth potential of the bacteria expressing the C-truncated enzymes is similar to that of bacteria expressing the full-length enzymes (Fig. 3).

The human and guinea pig L-asparaginases discussed in this work are homologous to the bacterial type I and type II L-asparaginases, respectively. However, whereas the bacterial enzymes contain only an L-asparaginase domain, the mammalian enzymes contain an additional C-terminal domain of ~200 residues of uncharacterized function. In order to understand the influence of the C-terminal domain on the catalytic activity of both hASNase1 and gpASNase1, we carried out protein truncation experiments. We first generated the C-truncated constructs for both enzymes leaving the proteins with only the N-terminal catalytic domain. We found that these C-truncated constructs retain activity as demonstrated by growth on minimal media similar to that of the full-length enzymes (Fig. 5). However, purification of these truncation variants revealed them to be relatively unstable, compelling us to work with the full-length versions.

Remember, to minimize immunogenicity against the L-asparaginase, we seek an enzyme that is as identical as possible to the human one but that has the low K_M_ property of the guinea pig/*E. coli* type II enzymes. The above experiments suggested that a chimera that has the guinea pig L-asparaginase domain followed by the human C-terminal domain would still retain the favorable kinetic properties of the gpASNase1 but would have a much increased sequence identity with the human enzyme.

To test this prediction, we generated two chimeras: one that we refer to as h_N_-g_C_ (human N-terminal domain fused to the guinea pig C-terminal domain) and the second, which is the inverse and called g_N_-h_C_ (Fig. 4). *In vitro* kinetic characterization of these chimeras validated the prediction, with h_N_-g_C_ displaying kinetic properties similar to hASNase1 and g_N_-h_C_ displaying kinetic properties similar to gpASNase1 (Table 1).

This result demonstrates that the C-terminal domain does not influence the catalytic activity of the L-asparaginase and most importantly does not negatively affect the K_M_. Since our goal is to identify a clone with gpASNase1 kinetic properties but with the highest sequence homology to hASNase1, we engineered the four clones isolated from the screen by replacing their shuffled C-terminal domain with the exact sequence of the human C-terminal domain (Fig. 4). As a result, the engineered clones, namely 63_N_-h_C_, 64_N_-h_C_, 65_N_-h_C_ and SA_N_-h_C_, display respectively 85.7%, 91.1%, 87.1% and 91.6% identity with the wild type hASNase1 sequence (Table 1 and Figure S6).

### Catalytic properties of the clones

For determining the precise kinetic properties of these four clones identified by the screen and comprising the C-terminal part of hASNase1, the genes were sub-cloned into a pET14b expression vector and expressed in C41 *E. coli* cells. The purified clones were tested for their L-asparaginase activity (see Table 1). *E. coli* L-asparaginase ansB was also included in order to compare our clones to an L-asparaginase approved for cancer therapy. We found that the four clones selected for by directed evolution and carrying the C-terminal domain of hASNase1 display high sequence identity with hASNase1 (>85%) but kinetic properties similar to gpASNase1 with a K_M_ in the micromolar range. Clone 63_N_-h_C_ (85.7% identity with the hASNase1 sequence) displays the lowest K_M_ at 47 μM. Clone SA_N_-h_C_ had the highest sequence identity to hASNase1 (91.6% identity), but this clone has a somewhat higher Asn K_M_ of 165 μM. We chose to compare the observed Asn hydrolysis rates of the enzymes at 50 μM Asn (k_obs@50µM_) as this is a relevant blood Asn concentration^4^. The *E. coli* ansB k_obs@50µM_ was found to be 41 ± 0.3 sec^−1^ and that of wild-type gpASNase1 was 20 sec^−1^. Importantly, the k_obs@50µM_ values for the humanized clones were also in this range, being 17 sec^−1^, 10 sec^−1^, 17 sec^−1^ and 6 sec^−1^ for clones 63_N_-h_C_, 64_N_-h_C_, 65_N_-h_C_ and SA_N_-h_C_, respectively.

### Cell culture evaluation of the humanized L-asparaginase clones

**Figure 6:**
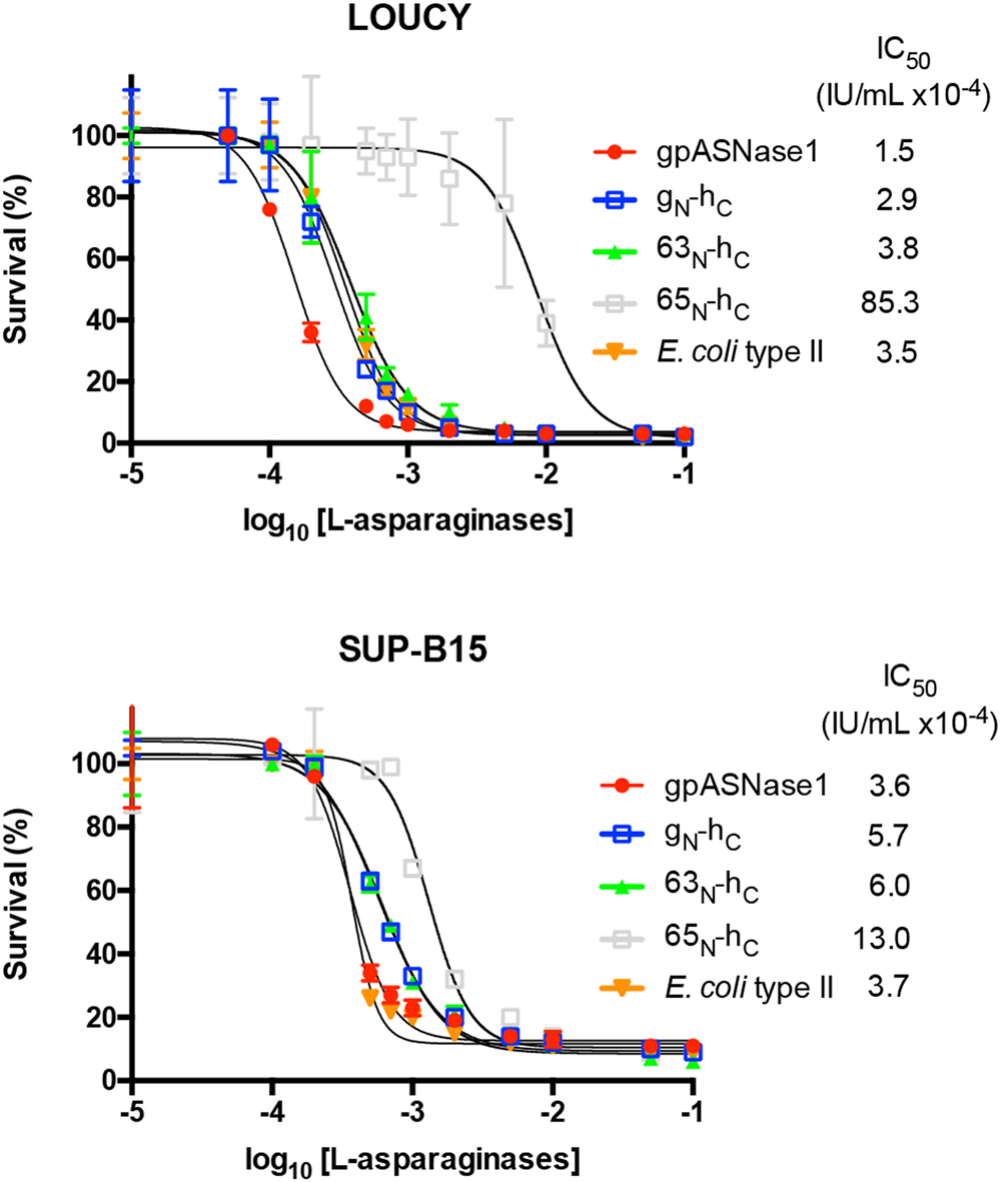
Human-like L-asparaginases clones possess cell-killing property in culture. The anticancer power of g_N_-h_C_ (blue box), 63_N_-h_C_ (green triangle) and 65_N_-h_C_ (open box), harboring the lowest Asn K_M_ values, were compared to the clinically relevant *E. coli* type II enzyme (orange triangle) and to gpASNase1 (red circle). A repeatable trend was found between the two tested ALL cell lines T-ALL LOUCY (upper panel) and B-ALL SUP-B15 (lower panel). While all tested L-asparaginases show good anticancer potency (IC_50_ in the mIU/mL range), g_N_-h_C_ and 63_N_-h_C_ exhibit very similar cell killing power, with an IC_50_ comparable to that of the *E. coli* type II enzyme and gpASNase1.

In our publication reporting the identification of a guinea pig L-asparaginase with micromolar K_M_ for Asn (i.e. gpASNase1), we made the prediction that this property will endow the enzyme with anti-ALL powers^11^. To test this prediction, we exposed the human T-ALL LOUCY and B-ALL SUP-B15 cell lines to increasing concentrations of gpASNase1. Indeed, as shown in Fig. 6, gpASNase1 exhibits an IC_50_ of 0.00015 IU/ml and 0.00036 IU/ml for the LOUCY and SUP-B15 cell lines, respectively. Notably, these IC_50_ values are comparable to those of the *E. coli* type II enzyme.

Next, having identified chimeras of hASNase1 and gpASNase1 whose K_M_ Asn is in the micromolar range, we evaluated the anti-ALL potency of three clones by comparing them to gpASNase1. We chose clones g_N_-h_C_, 63_N_-hC and 65_N_-hC for these experiments since these clones have the lowest Asn K_M_ (35, 47 and 74 µM, respectively) and thereby have activities most similar to that of gpASNase1 (50 µM) – Table 1. As expected, due to the higher K_M_ value of 65_N_-hC, this clone had the highest IC_50_ value compared to the other enzymes but was still very effective in killing both the T-ALL and B-ALL cells (IC_50_ in the mIU/mL range; Fig. 6). Clones g_N_-h_C_ and 63_N_-h_C_ proved to be very similar in their cell-killing power compared to gpASNase1. This is especially notable for clone 63_N_-h_C_, since this clone increases the percent identity to hASNase1 from 69.8% as present in gpASNase1 to 85.7%.

## Discussion

To overcome the immunogenicity problem related to the use of the *E. coli* L-asparaginase, attempts have been undertaken to de-immunize the bacterial enzyme^23–25^. Here we describe an alternative strategy that involves the development of a human-like L-asparaginase with kinetic properties similar to that of the type II *E. coli* L-asparaginase. Using a DNA family shuffling approach, we identified several clones with high sequence identity to hASNase1 but with the low K_M_ properties of the gpASNase1.

**Figure 7:**
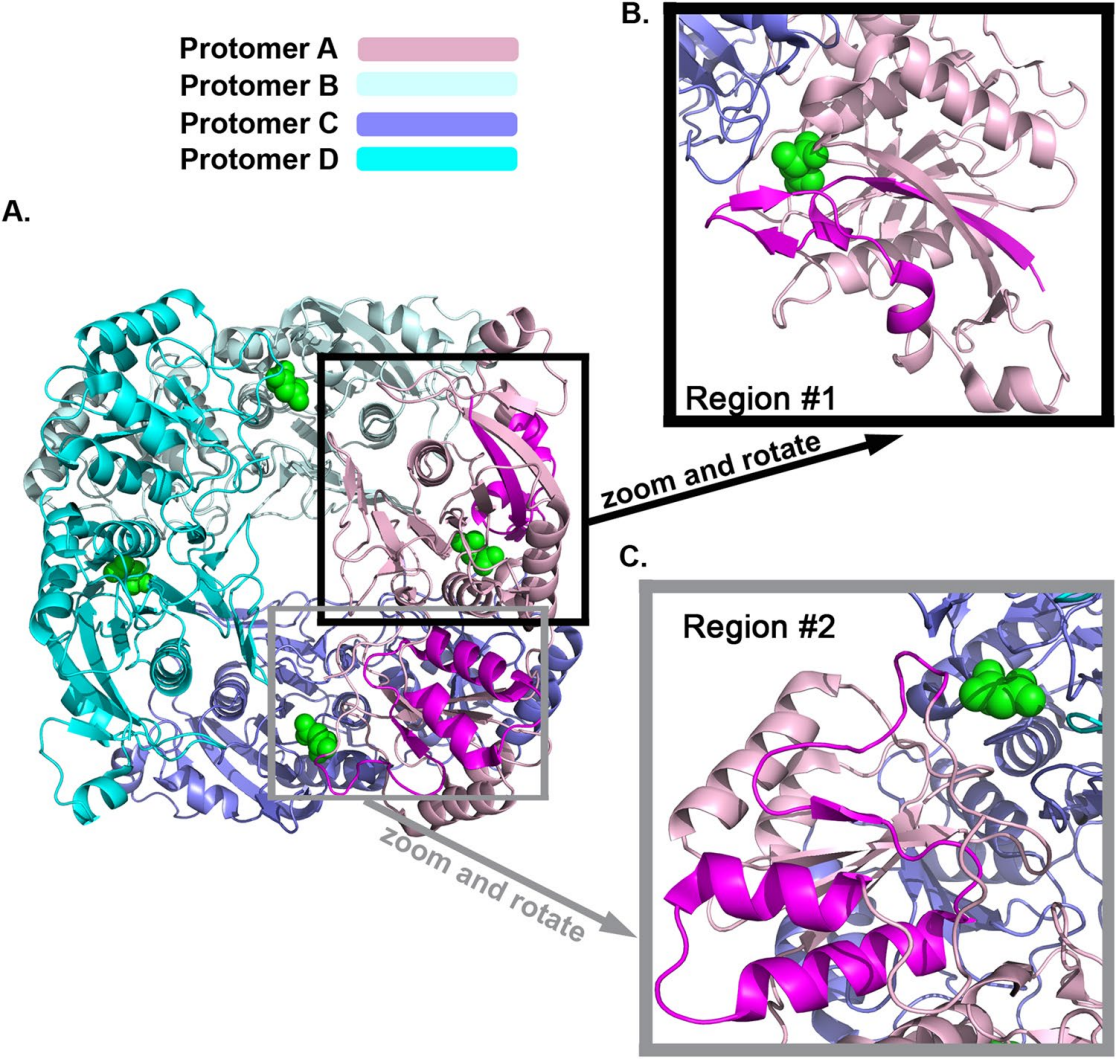
All low K_M_ chimera clones from the screen contained residues from gpASNase1 in regions #1 and #2. (**A**). Shown is a ribbon representation of the tetrameric gpASNase1 (PDB ID 4R8L), where each monomer is colored differently, the bound Asp reaction product is shown as green spheres, and regions #1 and #2 of protomer A colored in magenta. (**B**) Zoom on region #1. (**C**) Zoom on region #2. Note how region #1 is in close proximity to the active site of the same protomer, and region #2 is in close proximity to the active site of the adjacent protomer. Hence, both regions can play a role in the properties of the active site, thereby modulating the K_M_ property.

Interestingly, as depicted in Fig. 4, we noticed that all the selected clones maintain the gpASNase1 residues present in the N-terminus (residues 1–38; required-for-low-K_M_ region #1), and in a region near the C-terminus of the catalytic domain (residues 305–357, required-for-low-K_M_ region #2). For the goal of understanding the molecular reason behind the selection of these two regions in all low K_M_ clones, we analyzed our crystal structure of gpASNase1. Figure 7A shows the gpASNase1 tetramer where each protomer is colored differently. Note that this structure only includes the catalytic domain (residues 8–361), but since the missing C-terminal domain (residues 362–565) seems not to affect the kinetic parameters, all the determinants of the K_M_ property are present in this structure. The required-for-low-K_M_ regions #1 and #2 are highlighted in magenta in Fig. 7, and the active site bound product of the reaction, an Asp molecule, one in each protomer, is shown in green. Using this coloring scheme it becomes apparent that region #1 is in close proximity to the Asp molecule in the same protomer (Fig. 7B), and region #2 is in close proximity to the Asp molecule in the adjacent protomer (Fig. 7C). Therefore, both regions that comprise the active site of gpASNase1 must be intact in order to preserve its superior kinetic activity. The molecular mechanism behind this effect is still not clear and a part of active study. One possible mechanism that we are exploring is that the two-residue deletion present in region #2 in gpASNase1 compared to hASNase1 (Figure S6) may affect the dynamics of loop closure over the bound substrate. In addition, it is not clear whether or not both or either of the two regions on their own are sufficient and/or required to lead to an enzyme with a K_M_ in the micromolar range, and this too is currently under investigation.

The identification of relevant clones was made possible thanks to the selection system developed. In genetic complementation, the activity being investigated is intrinsically linked to the growth of the strain as the library complements the mutant strain. We describe in this paper two selection systems based on genetic complementation. We show that the strain BW2Δ was incapable of using Asn as a nitrogen source. Interestingly, we found that both the periplasmic (ansB) and cytoplasmic *E. coli* (ansA) L-asparaginases are involved in the use of Asn as a nitrogen source. Indeed, the single mutants BWΔansA and BWΔansB were not sufficient to individually impair the use of Asn as nitrogen source as it was previously described for a strain supposedly lacking the cytoplasmic enzyme^26^. However, this system could not be used for our study as it was not able to distinguish between low and high K_M_ L-asparaginase clones.

In contrast, we show that the BW5Δ *E. coli* strain can be used to select mutants harboring a low K_M_ L-asparaginase. Our observations are different to those of Cantor *et al*. who reported that they could not use this strain as a selection system due to the formation of colonies by null mutants, which they attributed to the spontaneous hydrolysis of Asn^23^. Using the minimal media described in the Methods section, we did not get any growth of the control strain BW5Δ pBAD even after incubation of the plates at 37 °C for several days. This suggests that under our conditions the amount of spontaneous Asn hydrolysis is not sufficient as an Asp source. In addition, in the absence of induction, BW5Δ pBAD_hASNase1 and BW5Δ pBAD_gpASNase1 could not grow. Upon induction, we were able to get colonies of homogenous size that were directly related to the activity of the enzyme as shown in Fig. 5. Although this selection system allowed us to isolate clones with the desired properties, one has to keep in mind that selection systems may be biased. In some cases, variants may be selected due to a higher expression level and not necessarily due to a higher enzymatic activity. Since we did not determine the expression level of the different L-asparaginases, we cannot rule out an effect due to differential expression. However, the fact that for a given concentration of inducer, both BW5Δ pBAD_hASNase1 and BW5Δ pBAD_gpASNase1 grew at the same speed at high concentration of Asn whereas only BW5Δ pBAD_gpASNase1 was capable of growing at low Asn concentration, is a strong indication that the difference in the ability to grow under low Asn conditions is related to the K_M_ property of the enzyme.

It is interesting to notice how directed evolution aiming to create new biocatalysts can help to generate variants that illuminate the relationship between enzyme sequence, structure, and function. Family shuffling is a powerful method that generates diversity by recombination, thereby combining useful regions from individual genes. Herein it was used to generate a chimera with the kinetic properties of the gene encoding gpASNase1 but with as much sequence homology as possible to the gene hASNase1. In this study, DNA shuffling turned out to be a more effective method for generating low K_M_ variants than the focused mutagenesis of active site residues. Although some of the libraries built by the CAST method contained mutations of residues located in region #1 (Figure S1), they were likely not sufficient to modulate hASNase1 property toward lower K_M_, hence suggesting again that both regions in gpASNase1 may be important to maintain low K_M_. Recently Karamitros *et al*. reported the use focused mutagenesis of four active site residues in hASNase3, another human L-asparaginase^27^. They report hASNase3 variants with 6-fold better catalytic efficiency (k_cat_/K_M_) as compared to the wild type enzyme. Recognizing that hASNase1 and 3 have much dissimilarity, it is interesting to note that the family shuffling protocol we used for hASNase1 allowed us to obtain a 140-fold increase in catalytic efficiency for clone #63_N_-h_C_. Moreover, this clone exhibits cell culture killing potency for ALL cells comparable to that of gpASNase1 (Fig. 6), thereby warranting further evaluation of the clinical potential of these human-like L-asparaginases.

This study also confirmed our prediction that gpASNase1 would possess an anti-ALL property. In addition, we observed that the C-terminal domains of gpASNase1 and hASNase1 could be swapped without significant change in the catalytic properties of the enzymes. Simply swapping the gpASNase1 C-terminal domain with that of hASNase1 (i.e. g_N_-h_C_) increases the identity to the human enzyme from 69.8 to 83.4%. This reduces the number of non-identical residues from 170 to 94. For clones 64_N_-h_C_ and SA_N_-h_C_ discovered by the screens, the percent identity increases to 91.1 and 91.6%, respectively, bringing the number of non-identical residues to <50 (Figure S6), however these clones had less favorable kinetics properties (i.e. higher K_M_) compared to the others. Clone 63_N_-h_C_ seems to possess the best combination of high identity to hASNase1 and *in vitro* cell killing power. Using 63_N_-h_C_ as a starting point, further reduction in non-human residues can now be potentially achieved by targeted mutagenesis, though it seems that multiple gpASNase1 residues (especially in regions #1 and #2) will need to be retained in order to maintain the required low Asn K_M_ property.

## Methods

### Strains

Chromosomal gene deletions were performed using the λ-red recombinase system^28^. The tyrosine aminotransferase gene *tyrB*, the aspartate aminotransferase gene *aspC* and the L-asparaginase genes *ansA*, *ansB, iaaA* were deleted from the chromosome of *E. coli* BW25113 F^-^, DE(araD-araB)567, lacZ4787(del)::rrnB-3, LAM^-^, rph-1, DE(rhaD-rhaB)568, hsdR514 resulting in *E. coli* BW5Δ. Briefly, a primer pair was used to amplify the gene replaced by the kanamycin resistance cassette from the appropriate keio strain as described by *Datsenko et al*.^28^. Subsequently, the linear PCR product was used to replace the entire ORF of the targeted gene on the BW25113 chromosome. Colonies containing the correct gene deletions were transformed with the FLP recombinase plasmid pCP20 to remove the kanamycin resistance marker, and the pCP20 was then cured from the resulting strain.

The strain *E. coli* BW2Δ was obtained following the same process and after the deletion of the genes *ansA* and *ansB*.

### Cloning of hASNase1 and gpASNase1 into BW2Δ and BW5Δ

The gene encoding the codon-optimized sequence of the hASNase1 (UNIPROT entry Q86U10) was amplified using the primers NdeI-hA_F1 and hA-BamHI_R573 and the codon-optimized sequence of gpASNase1 (UNIPROT entry H0W0T5) with the primers NdeI-gpA_F1 and gpA-BamHI_R565 (Table S1). After a BamHI/NdeI digestion, the PCR products were inserted into the pBAD vector. The resulting vectors were subsequently used to transform the strains BW5Δ and BW2Δ, resulting in the strains BW5Δ pBAD_hASNase1, BW5Δ pBAD_gpASNase1, BW2Δ pBAD_hASNase1 and BW2Δ pBAD_gpASNase1. BW5Δ and BW2Δ were also transformed with the empty pBAD vector to serve as controls.

### Media and growth experiments

M9 complete medium was made from M9 minimum salt (Sigma) supplemented with 0.4% glycerol, 2 μM thiamine, 1 mM MgSO_4_, 0.1 mM CaCl_2_ and 100 μg/mL ampicillin. For complete M9 plates, 15 g/L of agar was added. For the experiments with the strain BW2Δ, M9 medium was made without NH_4_Cl. When required, L-asparaginase was added to M9 medium at different concentrations. To induce the expression of hASNase1 and gpASNase1 cloned into the pBAD vector, 0.02% arabinose was added to the M9 medium. For the growth experiments on complete M9 on agar plates, the strains were first grown in LB overnight at 37 °C, spun down and washed in M9 medium. An appropriate dilution of this suspension was then spread on the M9 plates so as to get the same number of colonies for each strain. Plates were incubated at 37 °C for 48 h to 96 h.

### Random mutagenesis

Error prone PCR was performed either on the full-length sequence or on the C-truncated construct of hASNase1. PCR was carried out using 10 nM of template, 30 pmol of each of the forward and reverse primers, an unbalanced mix of dNTP (0.2 mM dATP and dGTP and 10 mM dTTP and dCTP), 8 mM MgCl_2_ and Taq polymerase (Promega). The amplified fragments were used as megaprimers to run a whole plasmid PCR^29^ using pBAD_hASNase1 as template. BW5Δ cells were transformed and plated on M9 plates supplemented with 0.2 mM Asn.

### Cast

The optimal choice of the respective pairs of amino acids was guided by the analysis of a homology model of hASNase1 generated from the crystal structure of gpASNase1. The following amino acid pairs or trios were defined: M22/R23, D84/S86, H114/G115, A142/Q143/V144, A191/R192, T118/F121, A91/C95/T99, R23/E25/L26 (Table S1). The 8 corresponding libraries were created separately using primers with codons displaying NNK degeneracy, used to transform BW5Δ cells and plated on M9 plates supplemented with 0.2 mM ASN.

### DNA shuffling

DNA shuffling was performed as described by Meyer *et al*.^30^ with slight adjustments. Briefly, an equimolar mixture of the hASNase1 and gpASNase1 genes were digested with 0.5 U of DNase (NEB) for 2 min 30 sec. Fragments between 100 bp and 200 bp were extracted using the Qiaquick gel extraction kit (Qiagen), reassembled by PCR and then amplified using either the primers specific to the gpASNase1 gene or the one specific to the hASNase1 gene. The obtained shuffled fragments were cloned into a pBAD vector using the Megawhop method^29^. Briefly, 100 to 300ng of shuffled fragments were used as megaprimers to run a whole plasmid PCR using either pBAD_hASNase1 or pBAD_gpASNase1 as template. After digestion of the template with DpnI, 20 to 40 ng of newly synthesized plasmids containing the shuffled sequences were used to transform electrocompetent BW5Δ cells. After the pulse, the cells were resuspended in 1 ml SOC medium and incubated with shaking at 37 °C for an hour. The cells were then spun down at 4000 x g for 4 min and gently resuspended in 200 μL of M9 medium. 100 μL were plated on M9 plate supplemented with 0.2 mM Asn or 2 mM Asn. After 4 days of incubation of the plates at 37 °C, the colonies from the 2 mM Asn plate were picked up and pooled into 200 μL of fresh M9 medium. 2 dilutions were successively carried out and used to plate fresh 0.2 mM ASN M9 plates. Clones capable of growing on the 0.2 mM Asn plate after 4 days of incubation at 37 °C and 3 additional days at room temperature were isolated and streaked on LB plates.

### C-terminal domain swapping

To build the clone h_N_-g_C_, the sequence corresponding to the N-terminal domain of hASNase1 (h_N_, residue 1–361) was amplified using the primers NdeI-hA_F1 and gpA360-367_R and the sequence corresponding to the C-terminal domain of gpASNase1 (gC, residue 360–565) was amplified using the primers hA354-361_F and gpA-BamHI_R565. To build the clone g_N_-h_C_ the sequence corresponding to the N-terminal domain of gpASNase1 (g_N_, residue 1–359) was amplified using the primers NdeI-gpA_F1 and hA362-369_R and the sequence corresponding to the C-terminal domain of hASNase1 (h_C_, residue 362–573) was amplified using the primers gpA352-359_F and hA_BamHI_R573. Primers are listed in Table S1. The chimera was then constructed by PCR fusion of fragment h_N_ and g_C_ or g_N_ and h_C_ using the appropriate primers and then subsequently cloned into the pBAD and pET vectors.

### Cloning and expression of the selected clones

The isolated clones were cultured; the plasmid was extracted and sequenced. The corresponding genes were transferred to a pET vector (modified pET14b to include a His-SUMO tag, using the same primers as the one used for cloning into the pBAD vector) to allow the expression of the His-tagged protein in C41 (DE3) cells. The culture was carried out in 1 L of 2YT medium supplemented with 100 μg/mL ampicillin. Expression was induced with 0.1 mM IPTG and cells were grown overnight at 18 °C. Cells were harvested, lysed and purified as previously described for the wild type gpASNase1^11^. The protein was eluted in a 25 mM Tris-HCl pH 7.5, 200 mM KCl, 500 mM imidazole buffer and dialyzed against the same buffer containing no imidazole but 1 mM of DTT. Expression and purification of the *E. coli* ansB enzyme was described previously^11^.

### Kinetic assays

The catalytic activity of the clones was determined using a spectroscopic NADH-dependent enzyme-coupled assay^31, 32^, which measures the production of Asp through the 1:1 oxidation of reduced NADH. The conversion of NADH to NAD was measured spectrophotometrically as a decrease in absorbance at 340 nm at 37 °C. All measurements were taken in triplicate in a buffer containing 100 mM Tris pH 7.5, 0.4 mM α-ketoglutarate and 0.4 mM NADH with 50 nM (hASNase1, hN-gC) 10 nM (gpASNase1, gN-hC, 63-hC, 64-hC, 65-hC, SA-hC) or 3 nM (ansB) enzyme. Glutamic-oxalacetic transaminase (Sigma G2751) and malic dehydrogenase (Sigma M2634) were helper enzymes for the coupled enzymatic reactions; 5 and 1 unit were used, respectively. Data were fit to the Michaelis–Menten equation using SigmaPlot (Systat Software Inc). Due to the cooperative nature of hASNase1, this enzyme was analyzed using the Hill equation.

### Cell culture

The LOUCY and SUP-B15 cell lines were kind gifts from Dr. Pieter Van Vlierberghe (Ghent University Hospital, Ghent, Belgium) and Dr. Michael Jensen (University of Washington School of Medicine, USA). All cell lines were analyzed by STR (Short Tandem Repeat) and confirmed to match 100% to corresponding STR profile data from the Global Bioresource Center ATCC. All cell lines were verified to be mycoplasma free. LOUCY and SUP-B15 lines were cultivated in a humid atmosphere (5% CO_2_, 37 °C) using RPMI 1640 media supplemented with 10% FBS (Hyclone) and 1x penicillin-streptomycin solution (Invitrogen). L-Glutamine was added directly into cell cultures to a final concentration of 2 mM. 90 μL aliquots of cell suspension (5 × 10^5^ cells per mL) were cultured in triplicate in round-bottomed 96-well microtiter plates in the presence of 10 μL of either DPBS (Dulbecco’s phosphate-buffered saline, Mediatech) or different L-asparaginases to a final concentration ranging from 0.00001 to 0.1 IU/mL. After incubating the plates for 4 days at 37 °C in humidified air containing 5% CO_2_, 11 μL of Alamar Blue (Invitrogen) was added to a final concentration of 10% (v/v) and the plates were incubated for an additional two hours, followed by reading of the fluorescence signal. The leukemic cell viability was calculated as percentage of fluorescence counts in the presence of L-asparaginase versus that in the DPBS control.

## Electronic supplementary material

Supplementary Data
