# Supplementary material for: Discovery of human-like L-asparaginases with potential clinical use by directed evolution
Source: Sci Rep. 2017 Aug 31;7:10224. doi: 10.1038/s41598-017-10758-4 (PMC5579231; doi:10.1038/s41598-017-10758-4)
Supplement: Supplementary file 1 — Supplementary Data [file 41598_2017_10758_MOESM1_ESM.docx]

**Supplementary Data**

**Discovery of human-like L-asparaginases with potential clinical use by directed evolution**

*Coraline Rigouin^1^, Hien Anh Nguyen^1,2^, Amanda M. Schalk^1^, and Arnon Lavie^1.2^**

^1^Department of Biochemistry and Molecular Genetics, University of Illinois at Chicago, Chicago, Illinois, United States of America

^2^The Jesse Brown VA Medical Center, Chicago, Illinois, United States of America

Running title: screen for low K_M_ L-asparaginases

*Address correspondence to: Arnon Lavie, PhD, 900 South Ashland Avenue, MBRB room 1108, Chicago, IL, 60607. Phone: (312) 355-5029; Fax: (312) 355-4535; Email: lavie@uic.edu

**Contents**

Figure S1: Residues selected for CAST

Figure S2: Amino acid sequence of hASNase1

Figure S3: Amino acid sequence of gpASNase1

Figure S4: DNA sequence of synthetic codon-optimized hASNase1

Figure S5: DNA sequence of synthetic codon-optimized gpASNase1

Figure S6: Amino acid sequence alignment of hASNase1, gpASNase1, and select humanized clones.

Table S1: List of the primers used in this study

**Figure S1: Residues selected for CAST on hASNase1 amino acid sequence**

**Residues selected for CAST mutagenesis in library 1 (turquoise), Lib2 (teal), Lib3 (yellow), Lib4 (green), Lib5 (red), Lib6 (blue), Lib7 (gray) and Lib8 (pink).**

MARAVGPERRLLAVYTGGTIGMRSELGVLVPGTGLAAILRTLPMFHDEEHARARGLSEDTLVLPPASRNQRILYTVLECQPLFDSSDMTIAEWVCLAQTIKRHYEQYHGFVVIHGTDTMAFAASMLSFMLENLQKTVILTGAQVPIHALWSDGRENLLGALLMAGQYVIPEVCLFFQNQLFRGNRATKVDARRFAAFCSPNLLPLATVGADITINRELVRKVDGKAGLVVHSSMEQDVGLLRLYPGIPAALVRAFLQPPLKGVVMETFGSGNGPTKPDLLQELRVATERGLVIVNCTHCLQGAVTTDYAAGMAMAGAGVISGFDMTSEAALAKLSYVLGQPGLSLDVRKELLTKDLRGEMTPPSVEERRPSLQGNTLGGGVSWLLSLSGSQEADALRNALVPSLACAAAHAGDVEALQALVELGSDLGLVDFNGQTPLHAAARGGHTEAVTMLLQRGVDVNTRDTDGFSPLLLAVRGRHPGVIGLLREAGASLSTQELEEAGTELCRLAYRADLEGLQVWWQAGADLGQPGYDGHSALHVAEAAGNLAVVAFLQSLEGAVGAQAPCPEVLPGV

**Figure S2:**

SEQ ID No1: hASNase1 amino acid sequence

MARAVGPERRLLAVYTGGTIGMRSELGVLVPGTGLAAILRTLPMFHDEEHARARGLSEDTLVLPPASRNQRILYTVLECQPLFDSSDMTIAEWVCLAQTIKRHYEQYHGFVVIHGTDTMAFAASMLSFMLENLQKTVILTGAQVPIHALWSDGRENLLGALLMAGQYVIPEVCLFFQNQLFRGNRATKVDARRFAAFCSPNLLPLATVGADITINRELVRKVDGKAGLVVHSSMEQDVGLLRLYPGIPAALVRAFLQPPLKGVVMETFGSGNGPTKPDLLQELRVATERGLVIVNCTHCLQGAVTTDYAAGMAMAGAGVISGFDMTSEAALAKLSYVLGQPGLSLDVRKELLTKDLRGEMTPPSVEERRPSLQGNTLGGGVSWLLSLSGSQEADALRNALVPSLACAAAHAGDVEALQALVELGSDLGLVDFNGQTPLHAAARGGHTEAVTMLLQRGVDVNTRDTDGFSPLLLAVRGRHPGVIGLLREAGASLSTQELEEAGTELCRLAYRADLEGLQVWWQAGADLGQPGYDGHSALHVAEAAGNLAVVAFLQSLEGAVGAQAPCPEVLPGV

**Figure S3:**

SEQ ID No2: gpASNase1 amino acid sequence

MARASGSERHLLLIYTGGTLGMQSKGGVLVPGPGLVTLLRTLPMFHDKEFAQAQGLPDHALALPPASHGPRVLYTVLECQPLLDSSDMTIDDWIRIAKIIERHYEQYQGFVVIHGTDTMASGASMLSFMLENLHKPVILTGAQVPIRVLWNDARENLLGALLVAGQYIIPEVCLFMNSQLFRGNRVTKVDSQKFEAFCSPNLSPLATVGADVTIAWDLVRKVKWKDPLVVHSNMEHDVALLRLYPGIPASLVRAFLQPPLKGVVLETFGSGNGPSKPDLLQELRAAAQRGLIMVNCSQCLRGSVTPGYATSLAGANIVSGLDMTSEAALAKLSYVLGLPELSLERRQELLAKDLRGEMTLPTADLHQSSPPGSTLGQGVARLFSLFGCQEEDSVQDAVMPSLALALAHAGELEALQALMELGSDLRLKDSNGQTLLHVAARNGRDGVVTMLLHRGMDVNARDRDGLSPLLLAVQGRHRECIRLLRKAGACLSPQDLKDAGTELCRLASRADMEGLQAWGQAGADLQQPGYDGRSALCVAEAAGNQEVLALLRNLALVGPEVPPAI

**Figure S4:**

SEQ ID No3: hASNase1 Synthetic codon-optimized gene(Genscript)

ATGGCTCGTGCTGTGGGTCCGGAACGCCGCCTGCTGGCTGTCTATACGGGTGGTACGATTGGTATGCGCTCTGAACTGGGTGTCCTGGTGCCGGGTACCGGTCTGGCAGCAATTCTGCGTACGCTGCCGATGTTTCATGATGAAGAACACGCACGTGCACGCGGTCTGTCGGAAGACACCCTGGTGCTGCCGCCGGCAAGCCGTAACCAGCGCATCCTGTATACCGTTCTGGAATGCCAACCGCTGTTTGATAGCTCTGACATGACCATTGCCGAATGGGTTTGTCTGGCACAGACGATCAAACGTCATTATGAACAATACCACGGTTTCGTGGTTATTCATGGCACCGATACGATGGCCTTTGCAGCTTCCATGCTGTCATTCATGCTGGAAAACCTGCAGAAGACCGTTATTCTGACGGGCGCTCAAGTCCCGATCCACGCACTGTGGAGCGACGGTCGCGAAAATCTGCTGGGCGCCCTGCTGATGGCAGGCCAGTATGTCATCCCGGAAGTGTGCCTGTTTTTCCAGAACCAACTGTTCCGTGGTAATCGCGCTACCAAAGTCGATGCGCGTCGCTTTGCGGCCTTCTGTAGCCCGAACCTGCTGCCGCTGGCTACCGTTGGTGCAGATATTACGATCAATCGTGAACTGGTGCGCAAAGTTGACGGCAAGGCAGGTCTGGTCGTGCATAGTTCCATGGAACAGGATGTCGGCCTGCTGCGTCTGTACCCGGGTATTCCGGCAGCTCTGGTGCGTGCCTTTCTGCAGCCGCCGCTGAAAGGTGTTGTCATGGAAACCTTCGGTTCTGGCAACGGTCCGACGAAGCCGGATCTGCTGCAGGAACTGCGTGTGGCAACCGAACGCGGCCTGGTCATTGTGAATTGCACGCACTGTCTGCAAGGCGCAGTTACCACGGATTATGCAGCCGGTATGGCAATGGCTGGTGCGGGTGTCATCTCGGGTTTTGACATGACCAGCGAAGCAGCTCTGGCGAAACTGTCCTACGTTCTGGGCCAGCCGGGTCTGTCACTGGATGTCCGTAAAGAACTGCTGACCAAGGACCTGCGCGGTGAAATGACGCCGCCGTCTGTGGAAGAACGTCGCCCGAGTCTGCAGGGTAACACCCTGGGCGGTGGCGTTTCTTGGCTGCTGTCACTGTCGGGCAGCCAAGAAGCCGATGCACTGCGCAATGCACTGGTGCCGAGTCTGGCATGCGCAGCAGCACATGCAGGTGACGTGGAAGCTCTGCAGGCGCTGGTTGAACTGGGTTCCGATCTGGGCCTGGTGGACTTTAACGGTCAAACCCCGCTGCATGCTGCAGCACGTGGTGGCCACACCGAAGCAGTTACGATGCTGCTGCAGCGTGGCGTTGATGTCAATACCCGCGATACGGACGGTTTCAGTCCGCTGCTGCTGGCAGTCCGTGGTCGTCATCCGGGCGTGATCGGTCTGCTGCGCGAAGCTGGTGCGTCTCTGAGTACCCAGGAACTGGAAGAAGCGGGCACGGAACTGTGTCGTCTGGCCTATCGCGCAGATCTGGAAGGCCTGCAAGTGTGGTGGCAAGCAGGTGCAGATCTGGGTCAGCCGGGTTACGACGGTCATAGCGCCCTGCACGTTGCAGAAGCAGCTGGTAATCTGGCTGTGGTTGCGTTTCTGCAAAGTCTGGAAGGTGCCGTCGGTGCCCAAGCGCCGTGCCCGGAAGTGCTGCCGGGTGTCTAA

**Figure S5:**

SEQ ID No4: gpASNase1 synthetic codon-optimized gene(Genscript)

ATGGCACGCGCTTCGGGCTCGGAACGTCATCTGCTGCTGATCTACACGGGTGGCACGCTGGGTATGCAATCCAAGGGTGGTGTTCTGGTCCCGGGTCCGGGTCTGGTGACCCTGCTGCGTACGCTGCCGATGTTTCATGATAAAGAATTCGCACAGGCACAAGGCCTGCCGGACCATGCTCTGGCGCTGCCGCCGGCCTCTCACGGTCCGCGCGTGCTGTATACCGTTCTGGAATGCCAGCCGCTGCTGGATAGCTCTGACATGACGATTGATGACTGGATTCGTATCGCGAAGATTATCGAACGCCATTATGAACAGTACCAAGGTTTTGTGGTTATCCACGGCACCGATACGATGGCTTCAGGCGCGTCGATGCTGAGCTTCATGCTGGAAAACCTGCACAAACCGGTGATTCTGACCGGTGCCCAGGTCCCGATCCGTGTGCTGTGGAACGATGCACGCGAAAATCTGCTGGGTGCCCTGCTGGTTGCAGGCCAGTATATTATCCCGGAAGTCTGCCTGTTTATGAACTCGCAACTGTTCCGTGGCAATCGCGTCACGAAGGTGGATAGCCAGAAATTTGAAGCATTCTGTTCTCCGAACCTGAGTCCGCTGGCTACCGTTGGTGCGGATGTCACGATTGCGTGGGACCTGGTTCGTAAAGTCAAGTGGAAAGATCCGCTGGTCGTGCATTCCAATATGGAACACGACGTCGCACTGCTGCGTCTGTACCCGGGCATCCCGGCTAGCCTGGTTCGTGCGTTTCTGCAGCCGCCGCTGAAGGGTGTTGTCCTGGAAACCTTCGGCTCCGGTAATGGCCCGTCAAAACCGGATCTGCTGCAGGAACTGCGTGCAGCAGCACAACGCGGCCTGATTATGGTGAACTGCTCGCAGTGTCTGCGCGGTAGCGTTACCCCGGGTTATGCCACGAGCCTGGCAGGTGCAAATATCGTGTCTGGCCTGGATATGACCAGTGAAGCTGCGCTGGCGAAGCTGTCTTACGTTCTGGGCCTGCCGGAACTGAGTCTGGAACGTCGCCAGGAACTGCTGGCTAAAGATCTGCGTGGTGAAATGACCCTGCCGACGGCAGACCTGCATCAGAGTTCCCCGCCGGGCTCCACCCTGGGTCAAGGCGTGGCCCGCCTGTTTTCACTGTTCGGTTGTCAGGAAGAAGATTCGGTGCAAGACGCAGTTATGCCGAGCCTGGCTCTGGCACTGGCACACGCAGGTGAACTGGAAGCCCTGCAAGCACTGATGGAACTGGGTTCCGATCTGCGTCTGAAAGACTCAAACGGCCAGACCCTGCTGCATGTTGCCGCACGTAATGGTCGCGATGGCGTGGTTACGATGCTGCTGCACCGCGGTATGGACGTGAACGCACGTGATCGTGACGGTCTGTCACCGCTGCTGCTGGCAGTTCAAGGTCGTCATCGCGAATGCATTCGTCTGCTGCGCAAGGCTGGCGCGTGTCTGTCTCCGCAGGATCTGAAAGACGCGGGTACCGAACTGTGCCGTCTGGCTAGTCGCGCGGATATGGAAGGTCTGCAGGCATGGGGTCAAGCAGGTGCAGATCTGCAGCAACCGGGTTACGACGGCCGTAGTGCCCTGTGTGTGGCAGAAGCTGCGGGTAATCAGGAAGTGCTGGCTCTGCTGCGTAATCTGGCTCTGGTTGGCCCGGAAGTCCCGCCGGCTATTTAA

**Figure S6: Sequence alignment of the clones tested for their kinetic properties**

gpASNase1 sequences are shaded green; hASNase1 sequences are without shading; Magenta bars correspond to the region #1 and #2 and are colored magenta in Figure 7 of the main text.

% identity

to hASNase1

100% hASNase1 MARAVGPERRLLAVYTGGTIGMRSELGVLVPGTGLAAILRTLPMFHDEEHARARGLSEDT

69.8% gpASNase1 MARASGSERHLLLIYTGGTLGMQSKGGVLVPGPGLVTLLRTLPMFHDKEFAQAQGLPDHA

86.5% hN-gC MARAVGPERRLLAVYTGGTIGMRSELGVLVPGTGLAAILRTLPMFHDEEHARARGLSEDT

83.4% gN-hC MARASGSERHLLLIYTGGTLGMQSKGGVLVPGPGLVTLLRTLPMFHDKEFAQAQGLPDHA

85.7% 63-hC MARASGSERHLLLIYTGGTLGMQSKGGVLVPGPGLVTLLRTLPMFHDKEFARARGLSEDT

87.1% 65-hC MARASGSERHLLLIYTGGTLGMQSKGGVLVPGPGLVTLLRTLPMFHDEEHARARGLSEDT

91.1% 64-hC MARASGSERHLLLIYTGGTLGMQSKGGVLVPGPGLVTLLRTLPMFHDEEHARARGLSEDT

91.6% SA-hC MARASGSERHLLLIYTGGTLGMQSKGGVLVPGPGLVTLLRTLPMFHDEEHARARGLSEDT

**** *.**.** :*****:**.*: ******.**.::*********:*.*.*.**.: :

hASNase1 LVLPPASRNQRILYTVLECQPLFDSSDMTIAEWVCLAQTIKRHYEQYHGFVVIHGTDTMA

gpASNase1 LALPPASHGPRVLYTVLECQPLLDSSDMTIDDWIRIAKIIERHYEQYQGFVVIHGTDTMA

hN-gC LVLPPASRNQRILYTVLECQPLFDSSDMTIAEWVCLAQTIKRHYEQYHGFVVIHGTDTMA

gN-hC LALPPASHGPRVLYTVLECQPLLDSSDMTIDDWIRIAKIIERHYEQYQGFVVIHGTDTMA

63-hC LVLPPASRNQRILYTVLECQPLLDSSDMTIDDWIRIAKIIERHYEQYQGFVVIHGTDTMA

65-hC LVLPPASRNQRILYTVLECQPLLDSSDMTIDDWIRIAKIIERHYEQYQGFVVIHGTDTMA

64-hC LVLPPASRNQRILYTVLECQPLFDSSDMTIAEWVCLAQTIKRHYEQYHGFVVIHGTDTMA

SA-hC LVLPPASRNQRILYTVLECQPLLDSSDMTIDDWIRIAKIIERHYEQYHGFVVIHGTDTMA

.*****.. *:**********:******* :*: :*: *:******:************

hASNase1 FAASMLSFMLENLQKTVILTGAQVPIHALWSDGRENLLGALLMAGQYVIPEVCLFFQNQL

gpASNase1 SGASMLSFMLENLHKPVILTGAQVPIRVLWNDARENLLGALLVAGQYIIPEVCLFMNSQL

hN-gC FAASMLSFMLENLQKTVILTGAQVPIHALWSDGRENLLGALLMAGQYVIPEVCLFFQNQL

gN-hC SGASMLSFMLENLHKPVILTGAQVPIRVLWNDARENLLGALLVAGQYIIPEVCLFMNSQL

63-hC FAASMLSFMLENLHKPVILTGAQVPIRVLWNDARENLLGALLVAGQYIIPEVCLFMNSQL

65-hC FAASMLSFMLENLHKPVILTGAQVPIHALWSDGRENLLGALLVAGQYIIPEVCLFMNSQL

64-hC FAASMLSFMLENLHKPVILTGAQVPIHALWSDGRENLLGALLMAGQYVIPEVCLFFQNQL

SA-hC FAASMLSFMLENLQKTVILTGAQVPIRVLWNDARENLLGALLVAGQYIIPEVCLFMNSQL

.***********:*.**********..**.*.*********:****:*******::.**

hASNase1 FRGNRATKVDARRFAAFCSPNLLPLATVGADITINRELVRKVDGKAGLVVHSSMEQDVGL

gpASNase1 FRGNRVTKVDSQKFEAFCSPNLSPLATVGADVTIAWDLVRKVKWKDPLVVHSNMEHDVAL

hN-gC FRGNRATKVDARRFAAFCSPNLLPLATVGADITINRELVRKVDGKAGLVVHSSMEQDVGL

gN-hC FRGNRVTKVDSQKFEAFCSPNLSPLATVGADVTIAWDLVRKVKWKDPLVVHSNMEHDVAL

63-hC FRGNRVTKVDSQKFEAFCSPNLSPLATVGADVTIAWDLVRKVKWKDPLVVHSNMEHDVAL

65-hC FRGNRVTKVDSQKFEAFCSPNLSPLATVGADVTIAWDLVRKVKWKDPLVVHSNMEHDVAL

64-hC FRGNRATKVDARRFAAFCSPNLLPLATVGADVTIAWDLVRKVKWKDPLVVHSSMEQDVGL

SA-hC FRGNRATKVDARRFAAFCSPNLSPLATVGADITINRELVRKVDGKAGLVVHSSMEQDVGL

*****.****:..* ******* ********:** .:*****. * *****.**:**.*

hASNase1 LRLYPGIPAALVRAFLQPPLKGVVMETFGSGNGPTKPDLLQELRVATERGLVIVNCTHCL

gpASNase1 LRLYPGIPASLVRAFLQPPLKGVVLETFGSGNGPSKPDLLQELRAAAQRGLIMVNCSQCL

hN-gC LRLYPGIPAALVRAFLQPPLKGVVMETFGSGNGPTKPDLLQELRVATERGLVIVNCTHCL

gN-hC LRLYPGIPASLVRAFLQPPLKGVVLETFGSGNGPSKPDLLQELRAAAQRGLIMVNCSQCL

63-hC LRLYPGIPASLVRAFLQPPLKGVVLETFGSGNGPSKPDLLQELRAAAQRGLIMVNCSQCL

65-hC LRLYPGIPASLVRAFLQPPLKGVVMETFGSGNGPTKPDLLQELRAAAQRGLIMVNCSQCL

64-hC LRLYPGIPAALVRAFLQPPLKGVVLETFGSGNGPSKPDLLQELRAAAQRGLIMVNCSQCL

SA-hC LRLYPGIPAALVRAFLQPPLKGVVMETFGSGNGPTKPDLLQELRVATERGLVIVNCTHCL

*********:**************:*********:*********.*::***::***::**

hASNase1 QGAVTTDYAAGMAMAGAGVISGFDMTSEAALAKLSYVLGQPGLSLDVRKELLTKDLRGEM

gpASNase1 RGSVTPGYAT--SLAGANIVSGLDMTSEAALAKLSYVLGLPELSLERRQELLAKDLRGEM

hN-gC QGAVTTDYAAGMAMAGAGVISGFDMTSEAALAKLSYVLGQPGLSLDVRKELLTKDLRGEM

gN-hC RGSVTPGYAT--SLAGANIVSGLDMTSEAALAKLSYVLGLPELSLERRQELLAKDLRGEM

63-hC RGSVTPGYAT--SLAGANIVSGLDMTSEAALAKLSYVLGLPELSLERRQELLAKDLRGEM

65-hC RGSVTPGYAT--SLAGANIVSGLDMTSEAALAKLSYVLGLPELSLERRQELLAKDLRGEM

64-hC RGSVTPGYAT--SLAGANIVSGLDMTSEAALAKLSYVLGLPELSLERRQELLAKDLRGEM

SA-hC QGAVTTGYAT--SLAGANIVSGLDMTSEAALAKLSYVLGLPELSLERRQELLAKDLRGEM

.*:**..**: ::***.::**:**************** * ***: *:***:*******

hASNase1 TPPSVEERRPSLQGNTLGGGVSWLLSLSGSQEADALRNALVPSLACAAAHAGDVEALQAL

gpASNase1 TLPTADLHQSSPPGSTLGQGVARLFSLFGCQEEDSVQDAVMPSLALALAHAGELEALQAL

hN-gC TLPTADLHQSSPPGSTLGQGVARLFSLFGCQEEDSVQDAVMPSLALALAHAGELEALQAL

gN-hC TPPSVEERRPSLQGNTLGGGVSWLLSLSGSQEADALRNALVPSLACAAAHAGDVEALQAL

63-hC TPPSVEERRPSLQGNTLGGGVSWLLSLSGSQEADALRNALVPSLACAAAHAGDVEALQAL

65-hC TPPSVEERRPSLQGNTLGGGVSWLLSLSGSQEADALRNALVPSLACAAAHAGDVEALQAL

64-hC TPPSVEERRPSLQGNTLGGGVSWLLSLSGSQEADALRNALVPSLACAAAHAGDVEALQAL

SA-hC TPPSVEERRPSLQGNTLGGGVSWLLSLSGSQEADALRNALVPSLACAAAHAGDVEALQAL

* *:.: ...* *.*** **:.*:** *.** *::.:*::**** * ****::******

hASNase1 VELGSDLGLVDFNGQTPLHAAARGGHTEAVTMLLQRGVDVNTRDTDGFSPLLLAVRGRHP

gpASNase1 MELGSDLRLKDSNGQTLLHVAARNGRDGVVTMLLHRGMDVNARDRDGLSPLLLAVQGRHR

hN-gC MELGSDLRLKDSNGQTLLHVAARNGRDGVVTMLLHRGMDVNARDRDGLSPLLLAVQGRHR

gN-hC VELGSDLGLVDFNGQTPLHAAARGGHTEAVTMLLQRGVDVNTRDTDGFSPLLLAVRGRHP

63-hC VELGSDLGLVDFNGQTPLHAAARGGHTEAVTMLLQRGVDVNTRDTDGFSPLLLAVRGRHP

65-hC VELGSDLGLVDFNGQTPLHAAARGGHTEAVTMLLQRGVDVNTRDTDGFSPLLLAVRGRHP

64-hC VELGSDLGLVDFNGQTPLHAAARGGHTEAVTMLLQRGVDVNTRDTDGFSPLLLAVRGRHP

SA-hC VELGSDLGLVDFNGQTPLHAAARGGHTEAVTMLLQRGVDVNTRDTDGFSPLLLAVRGRHP

:****** * * **** **.***.*. .*****:**:***:** **:*******.***

hASNase1 GVIGLLREAGASLSTQELEEAGTELCRLAYRADLEGLQVWWQAGADLGQPGYDGHSALHV

gpASNase1 ECIRLLRKAGACLSPQDLKDAGTELCRLASRADMEGLQAWGQAGADLQQPGYDGRSALCV

hN-gC ECIRLLRKAGACLSPQDLKDAGTELCRLASRADMEGLQAWGQAGADLQQPGYDGRSALCV

gN-hC GVIGLLREAGASLSTQELEEAGTELCRLAYRADLEGLQVWWQAGADLGQPGYDGHSALHV

63-hC GVIGLLREAGASLSTQELEEAGTELCRLAYRADLEGLQVWWQAGADLGQPGYDGHSALHV

65-hC GVIGLLREAGASLSTQELEEAGTELCRLAYRADLE-------------------------

64-hC GVIGLLREAGASLSTQELEEAGTELCRLAYRADLEGLQVWWQAGADLGQPGYDGHSALHV

SA-hC GVIGLLREAGASLSTQELEEAGTELCRLAYRADLEGLQVWWQAGADLGQPGYDGHSALHV

* ***:***.**.*:*::********* ***:*

hASNase1 AEAAGNLAVVAFLQSLEGAVGAQAPCPEVLPGV

gpASNase1 AEAAGNQEVLALLRNL-ALVG-----PEVPPAI

hN-gC AEAAGNQEVLALLRNL-ALVG-----PEVPPAI

gN-hC AEAAGNLAVVAFLQSLEGAVGAQAPCPEVLPGV

63-hC AEAAGNLAVVAFLQSLEGAVGAQAPCPEVLPGV

65-hC ---------------------------------

64-hC AEAAGNLAVVAFLQSLEGAVGAQAPCPEVLPGV

SA-hC AEAAGNLAVVAFLQSLEGAVGAQAPCPEVLPGV

**Table S1: List of primers**

| Primer name | Sequence 5’ – 3’ |
| --- | --- |
| gpA-BamHI_R565 | AACGTCGGATCCTTAAATAGCCGGCGGGACTTC |
| gpA_F | ATGGCACGCGCTTCGGGCTCGGAA |
| gpA_R | CCAGATTACGCAGCAGAGC |
| hA-F | GCTCGTGCTGTGGGTCCGGAA |
| hA_R | GACACCCGGCAGCACTTC |
| gpA360-367_R | CTGATGCAGGTCTGCCGTCGGCAGCGTCATTTCACCGCGCAGGT |
| hA354-361_F | AAGGACCTGCGCGGTGAAATGACGCTGCCGACGGCAGACCTGC |
| hA362-369_R | GCGACGTTCTTCCACAGACGGCGGGGTCATTTCACCACGCAGATC |
| gpA352-359_F | AAAGATCTGCGTGGTGAAATGACCCCGCCGTCTGTGGAAGAACG |
| Lib1 M22R23_F | TACACCGGCGGCACCATTGGCNNKNNKAGTGAGCTCGGCGTGCTTGTG |
| Lib1 M22R23_R | CACAAGCACGCCGAGCTCACTMNNMNNGCCAATGGTGCCGCCGGTGTA |
| Lib2 D84S86_F | CTGGAGTGCCAGCCCCTCTTCNNKTCCNNKGACATGACCATCGCTGAGTGG |
| Lib2 D84S86 R | CCACTCAGCGATGGTCATGTCMNNGGAMNNGAAGAGGGGCTGGCACTCCAG |
| Lib3 H114G115_F | CAGTACCACGGCTTTGTGGTCATCNNKNNKACCGACACCATGGCCTTTGCTGCC |
| Lib3 H114G115_R | GGCAGCAAAGGCCATGGTGTCGGTMNNMNNGATGACCACAAAGCCGTGGTACTG |
| Lib4 A142Q143V144_F | AGAAGACTGTCATCCTCACTGGGNNKNNKNNKCCCATCCATGCCCTGTGGAGC |
| Lib4 A142Q143V144_R | GCTCCACAGGGCAT GATGGGMNNMNNMNNCCCAGTGAGGATGACAGTCTTCT |
| Lib5 A191R192_F | GCAACCGGGCAACCAAGGTAGACNNKNNKAGGTTCGCAGCTTTCTGCTCCCC |
| Lib5 A191R192_R | GGGGAGCAGAAAGCTGCGAACCTMNNMNNGTCTACCTTGGTTGCCCGGTTGC |
| Lib6 T118F121_F | TCATCCACGGCACCGACNNKATGGCCNNKGCTGCCTCGATGCTGTCC |
| Lib6 T118F121_R | GGACAGCATCGAGGCAGCMNNGGCCATMNNGTCGGTGCCGTGGATGA |
| Lib7 A91C95T99_F | TCCAGTGACATGACCATCNNKGAGTGGGTTNNKCTTGCCCAGNNKATCAAGAGGCACTACGAG |
| Lib7 A91C95T99_R | CTCGTAGTGCCTCTTGATMNNCTGGGCAAGMNNAACCCACTCMNNGATGGTCATGTCACTGGA |
| Lib8 R23E25L26_F | GGCGGCACCATTGGCATGNNKAGTNNKNNKGGCGTGCTTGTGCCCGGG |
| Lib8 R23E25L26_R | CCCGGGCACAAGCACGCCMNNMNNACTMNNCATGCCAATGGTGCCGCC |
